# Less Is More: Norwegian Drug Regulation, Antibiotic Policy, and the “Need Clause”

**DOI:** 10.1111/1468-0009.12405

**Published:** 2019-07-21

**Authors:** BÅRD HOBÆK, ANNE KVEIM LIE

**Affiliations:** ^1^ TIK‐Centre, Faculty of Social Sciences University of Oslo; ^2^ Institute of Health and Society, Faculty of Medicine University of Oslo

**Keywords:** drug regulation, pharmaceutical policy, antibiotic resistance, need clause

## Abstract

**Policy Points**

- The current crisis of antibiotic resistance calls for policy reforms locally and globally. Historical insight in different regulatory systems can inform current decision making.
- A strong regulatory control implementing antimicrobial resistance concerns can ensure the combined objective of promoting access and limiting excess use by letting only certain drugs onto the market in compliance with public health needs.
- Regulation at this level also has powerful effects on consumption and needs to be considered as a tool for curbing antibiotic resistance.
- The Norwegian drug regulatory procedures was an example of how national drug regulatory authorities can promote innovation of new drugs that meet public health needs indirectly by accepting only drugs of added therapeutic value.

**Context:**

Antibiotic resistance is an increasingly serious threat to global health that requires coordinated action. Most current policy efforts address the lack of medicines. There is also a need for new thinking on promoting access to all who are in need of antibiotics, while simultaneously curbing inappropriate use. As the situation calls for new approaches, we examined one drug regulatory system in which antimicrobial resistance (AMR) has been on the agenda for a long time. The Norwegian drug regulatory system, and particularly its “need clause,” has been invoked in international debates but not previously studied in detail.

**Methods:**

We conducted a historical review of the Norwegian drug regulatory system by examining the archives of the Norwegian health authorities, the Norwegian Medicines Agency, and policy debates in the period.

**Findings:**

The Norwegian drug regulatory system focused on the rational use of drugs, tied closely to public health needs. It was originally written to address unnecessary consumption of drugs, not consumer protection and safety. The most flexible element within this system stated that a drug must be “needed” in order to be registered. When antibiotic resistance became a concern, it limited the market entry of drugs considered to promote resistance, such as combination and broad‐spectrum products. This was a powerful and flexible regulatory device that also influenced drug consumption.

**Conclusions:**

The need clause has lately been promoted as an alternative to address the current situation. The solutions to the problem of antibiotic resistance cannot be the same everywhere, and we do not argue that this drug regulatory system should be adopted globally. However, the current situation calls for consideration of many different aspects. This historical case demonstrates how regulatory procedures can be used to limit market entrance and promote appropriate use simultaneously.

Antibiotic resistance is increasingly acknowledged as a global health emergency, requiring concerted global action on the levels of drug production, drug regulation, and drug stewardship.^1^, ^2^ There is no doubt that antibiotic consumption is a main driver of selection pressure that contributes to resistance. Antibiotic use is related to incentives and behavior of patients, providers, producers, payers, and policymakers. The balance between securing access to life‐saving antibiotics for all who need them while at the same time restricting overuse that contributes to resistance is difficult to achieve. The Norwegian drug regulatory system in the 20th century succeeded in doing both. This system has been cited as a factor in explaining the country's historically lower incidence of antibiotic resistance compared to most other countries^3^ and has recently received scholarly attention in the context of current debates over drug regulation.^4^, ^5^, ^6^ Norway and Sweden were among the first countries to develop drug regulations related to safety and efficacy in the 1920s and 1930s. An assessment of relative need for a drug—the so‐called need clause—was included in the legal requirement for a drug to obtain access to the Norwegian market, resulting in very limited national formularies.^7^, ^8^, ^9^, ^10^, ^11^, ^12^, ^13^, ^14^ In this article, we discuss the history of the Norwegian comprehensive drug regulatory system in the 20th century. We focus on the changing roles, justifications, and specific uses of the need clause in connection with a strict system of regulating the use of antibiotics, as antimicrobial resistance gradually emerged as a serious problem for public health.

This system was originally designed to address the perceived societal problem of the entry of irrational or unnecessary drugs into the marketplace, with all of the irrational and unnecessary use and consequences that would follow. It was not explicitly framed in terms of consumer protection or safety, as was predominant elsewhere. This remained the focus all through the so‐called therapeutic revolution, where a new research‐based and prolific pharmaceutical industry started to pour new and profitable drugs onto the market.^15^ The new “wonder drugs,” such as antibiotics, were hailed as breakthroughs, but at the same time seen as precious and in need of protection against overuse and market forces. When antibiotic resistance came to be seen as a serious challenge in the 1970s, the need clause turned out to be a very useful legal measure.

The concept of “need” was never defined in the law and was thus open to interpretation and could include a number of different public health considerations. Earlier accounts have discussed the need clause almost exclusively in terms of simply reducing the numbers of drugs available. We argue, however, that equally important was how it provided an opportunity to establish a wide maneuvering space to pursue specific regulatory agendas, with restrictive antibiotics policy as one of the most clear‐cut cases.

## Context of Origin: Containing “a Flood of Drugs”

To grasp important specificities of the Norwegian drug regulatory system, it is necessary to go back to its beginnings. The main framework of this system was established already in 1928. Key features of the system were shared with the other Nordic countries, whose drug laws are routinely cited as the earliest of their kind.^13^, ^16^, ^17^, ^18^, ^19^, ^20^ It established the principle that a drug had to be approved of before it could be legally sold—that is, preapproval, also referred to as registration, market authorization, or licensing. Sweden imposed this regulation in 1935, while Denmark introduced a system of sampling to check composition, quality, and declared contents in the 1930s and preapproval in 1954.^21^ The Norwegian regulation of 1928 was designed to control “specialties,” or manufactured drugs, as opposed to drugs compounded in the individual pharmacy. In contrast to the US 1906 Pure Food and Drug Act, which prescribed penalties for misbranded and adulterated drugs already on the market, the Norwegian law demanded that any drug or “specialty” had to be registered and approved *before* it could be marketed. Approval was granted on the basis of a number of requirements. The drug's name, composition and price, the purity and quality of its ingredients, its label, and its promotional material all had to be evaluated before approval could be made. Most important, the drug had to be “medically justified,” have a price in proportion to value, and be appropriately advertised. A new testing laboratory was established to review the drug applications, and this new regulatory apparatus gradually expanded its activities in the 1930s. From 1935, approval was limited to a five‐year period.

These new regulations—in clear contrast to later processes in the United States and Europe—met very little opposition in the preparatory, legislative process.^22^, ^23^, ^24^ Premarket review and notification, withdrawal authority, labeling regulation, and compulsory disclosure of all drug contents—what Daniel Carpenter has called the “four enumerated powers,”—were already in place in Norway in the 1930s.^25^ ^(pp100‐101)^ These appear to have been thought of as necessary measures to limit a mass of dubious compounds at a time when there had been no established apparatus to verify the declarations or regulate extravagant claims made in advertisements. The legislative preparatory documents declared that pharmacists and physicians stood “defenseless” against a rising “flood of bogus drugs.” The main target of the new law was the pharmaceutical industry's “lower ranks,” the “parasites” and “weeds in its field,” the swindlers whose “only guideline is that sick people are excellent for purposes of commercial exploitation.”^22^ ^(pp8‐12)^

This original orientation of the regulatory system against irrational use of heavily promoted products is very different from the paradigmatic cases in the history of drug regulation. Safety, risk, or side effects were not among the explicitly mentioned criteria for evaluation and approval. In contrast, safety considerations were crucial in shaping and triggering drug regulation in many other countries, most famously in the case of the United States, where the sulfanilamide disaster of 1937 that killed more than 100 people was crucial to the Federal Food, Drug, and Cosmetic Act of 1938. Likewise, the thalidomide disaster of the late 1950s and early 1960s, where more than 10,000 children worldwide were born with malformations as a result of their mothers’ intake of the drug in pregnancy, led to a string of drug regulatory initiatives worldwide, most of them patterned on the American model.^25^, ^26^ Although misleading claims in advertising was also part of this American model's beginnings, safety was clearly the central issue in the development of modern drug regulation. In the Norwegian case, explicit safety concerns came to be included gradually along with efficacy within the larger notion of “medically justified” drugs. This followed naturally from the perceived problem the legislation was designed to solve, namely, the flood of highly advertised but inferior or useless drugs, leading to wasteful, misguided use—in both medical and economical terms. In fact, American regulatory officials looked to Swedish and Norwegian legal precedents when they tried to include efficacy requirements in the premarket review authority in 1937 and 1938. Although they did not succeed, US Food and Drug Administration (FDA) officials continued to study the regulatory laws in the Nordic countries throughout the 20th century.^25^ ^(p137)^

A formal requirement that a drug must be “needed” to be approved was included in a revision of the law in 1938. Surprising as it may seem to us today, this additional normative requirement—that a drug be “medically justified *and needed*,”—was hardly even mentioned in the parliamentary process leading up to the revision.^27^, ^28^, ^29^ Neither was it a matter of controversy in the press. The requirement for assessment for relative need has been regarded in retrospect as a very radical measure. However, at the time it was seen more as a logical extension of the established system in which the perceived danger was a “flood” of dubious drugs. Seen in this light, “need” was already in accordance with the way “medically justified” had been interpreted in the preceding years, in the practice established by the new registration authority.

From then on, it was not enough for a drug to be efficacious; it had to have a relative therapeutic value in relation to other products already on the market. In other words, a new drug should fill a need not already met by currently marketed alternatives, or do so in a better way, such as a lower price. In practice, this translated into an opportunity for the registration authority to limit the number of available drugs on the market, in terms of both chemical entities and different brands and dosage forms. It was used actively to maintain one of the smallest numbers of available drugs on the market in the world. The total number of drugs, however, is not the whole story about the effects of the need clause; it has also been tied closely to notions of rational use, and more specifically, to less prescription of antibiotics. As we argue in the following pages, there were many ways that the need clause could shape antibiotic prescribing, far beyond regulating the simple total number of drugs available. The restrictive market access was a part of and helped constitute a broader ecological and public health perspective with respect to the consequences of drug use in general and antibiotic use in particular and to a more conservative usage of the drugs already on the market.

## Penicillin and the Need Clause in Practice

The need clause first came into force in 1941, and the detailed regulations did not specify the notion of need. It was left up to the health director to work out the organization of the control and the criteria for registration.^30^ Karl Evang was director of health from 1938 to 1972, with five years in government exile during World War II, and he actively fought to secure drug policy as first and foremost an integral part of a general health and social policy, *not* a matter of industrial or commercial policy.^31^ He quickly established a strict interpretation of how “need” was to be evaluated in the postwar years. The health director had the authority of approval for drugs, and this was not just a formal authority. Evang appointed a council of trusted physicians and pharmacists and personally led discussions and made the final decisions for each drug in monthly meetings until his retirement in 1972. After a revision of the law in 1964, this authority was shared with an expanded Specialties Board (*Spesialitetsnemnda*) of five members, headed by the health director.

Overuse of antibiotics was one of the problem areas that Evang singled out early on. According to him, overuse had quickly “dulled the new sword put in our hands.” In the late 1940s, he received harsh public criticism for the restrictive policy the Norwegian health authorities had adopted on certain antibiotics.^32^ In 1964, however, he could in retrospect take pride in a significantly smaller problem of resistance development in his own country than elsewhere.^33^

This at first appears a little strange, since the registration authority was not skeptical toward antibiotics. During the first decades after the war, the new antibiotics were generally welcomed as important breakthroughs, and they were approved for marketing without much further ado. Penicillin was even mentioned specifically as an example of a drug for which the need clause was *not* practiced as usual, allowing 11 brands to be marketed simultaneously, as opposed to the normal two or three.^34^ But the important difference when compared to, for instance, the US system was not only the *number* of brands on the market; it was also *what kind of antibiotics* that could be excluded by the need clause. Already in 1947, a euphoric moment after the development of penicillin,^35^ Evang and his expert council were debating whether the miracle of penicillin would be a temporary one. As penicillin was becoming readily available, they worried that its overuse could lead to development of resistant bacteria, as had happened with the sulfa drugs.

The immediate pretext for the discussion was the application in 1946 for registration of penicillin in new forms that to the health authorities did not seem strictly necessary—such as pastilles or cough drops. Development of resistance had not to their knowledge been clearly shown clinically, but like Alexander Fleming, they saw it as a real possibility.^36^ “Sensitization”—or development of allergic reactions—to penicillin was, however, perceived as a real problem, and this was discussed in parallel with the resistance issue in Evang's expert council, as well as in the main journal of the Norwegian Medical Association.^37^ Converging lessons could be drawn from both problems: the rapidly increasing consumption of easily available penicillin was irrational and could seriously undermine its value.

Considerations such as these had initially led to the denial of approval for several penicillin products, “because of the danger that their use could lead to development of penicillin‐resistant strains of microbes.”^38^ A Danish doctor whom Evang had asked to give his views on resistance development replied that although reports were still inconclusive, precautionary measures should be taken. The scenario of “a population uncritically munching penicillin” should be avoided in any case, as should any temptation for doctors to replace the proven and rational treatment by injection with convenient and dubious alternatives. The drugs in question were reluctantly approved, but were limited to a one‐time‐only prescription, thus curbing the potential for widespread use.^38^ As a general rule, it was quickly established that antibiotics were available only by prescription, not for over‐the‐counter use.

Important features of the Norwegian antibiotic policy and drug regulation can already be seen in this early episode. Questions of “irrational” drug use and overuse were an integral part of the regulatory framework in which drugs were assessed. Hence, indirect and societal problems resulting from unnecessary use formed an integral part of the initial judgment by the registration authorities, instead of being simply a question of responsible use once the drug was available on the market. It was not sufficient to document safety and efficacy to be granted the right to market a drug, as gradually came to be the requirements internationally in the decades following the war. Instead, drugs could be denied approval or restricted if they did not merit inclusion in the preferred overall therapeutic regime, where a wider range of considerations would be taken into account. In this case, penicillin was perhaps the most highly valued drug available and still its use could be limited by restricting availability and dosage forms other than those deemed appropriate. Thus, the already established framework of drug regulation provided readily available means to address concerns such as the still only hypothetical problem of penicillin resistance. Decisions such as these took part in maintaining a higher barrier for use, before prescription patterns and patient expectations had been formed. It thus provided a path for public health concerns to find their way directly into the formation of habits and expectation of prescription—in this case, a more conservative usage of antibiotics.

Another important characteristic is that the distance between regulatory action and expert opinions and concerns was often very short, and this remained the case up until the 1980s. In this particular case, the health director took the initiative himself, soliciting external expert opinion and discussing restrictions even before resistance had been proven as a problem. The use of external expert opinion is of course anything but unique to the Norwegian case, but the translation into regulatory action was often very quick when the leadership was in agreement. Whereas the FDA waited until the end of the 1960s to introduce efficacy requirements with the consequent removal of fixed‐dose combination antimicrobials,^39^, ^40^ the need clause could be used to exclude combination drugs from the outset. The Norwegian health authorities maintained an outspoken skeptical attitude toward combination drugs. When promoted “without regard for therapeutic principles,” they were “at best fraudulent and at worst dangerous,” the head of the pharmaceutical division between 1964 and 1991 asserted in retrospect.^11^ ^(p83)^ Already in 1945, the control agency reported to have rejected drugs that had an “irrational composition,” that turned out be “superfluous combinations.” As a matter of principle, the agency was skeptical of prefixed combinations. Later guidelines for the application process underlined that combination drugs would “require a very good justification.”^41^ The ability to keep drugs such as these off the market with a simple “no need” was foregrounded as one of the key advantages of the need clause.

Along with a restrictive policy on dosage forms and over‐the‐counter availability, this limited the formation of the antibiotic consumption patterns elsewhere singled out as the most problematic in the postwar years: uncritical and nonspecific use, often in cases where the drug would have limited or no effect, and where drug combinations with broad‐spectrum antibiotics substituted for proper diagnosis. Broad‐spectrum antibiotics were also repeatedly met with suspicion, particularly from the late 1960s. In fact, narrow‐spectrum antibiotics continue to be much used in Norway to this day.

## Rational Therapeutics and the Logic of the Market

Drug policy in Scandinavia in the postwar years was influenced by the American notion of “rational therapeutics.” From the early 1900s this notion had an anticommercial sentiment, and this heightened after World War II.^42^ Irrational therapeutics came to be seen as driven by advertisers and demanding patients.^43^, ^44^ The proponents of rational medicine saw physicians as at the mercy of advertisers and patients wanting new drugs. Along with the increasing pace of development of medical practice, this made the physicians easy prey for industry.

In the United States, Harry Dowling and Maxwell Finland famously promoted controlled clinical trials as the most important tool to achieve rational therapy. In Scandinavia, the main proponents of rational medicine also worked across other arenas: within different parts of the public health system, as bureaucrats, regulators, policymakers, or affiliated as advisers. Many of the staff in the Health Directorate, like the health director himself, Karl Evang, had a degree in public health from Johns Hopkins University. Courses based on American public health approaches were arranged for employees in the Health Directorate, and arguments in favor of restrictive use found easy resonance in the “medicratic” administration.^45^

Administrative officials saw commercial pressure from the pharmaceutical industry on physicians and patients, and the consequent overuse of drugs, as a threat both to public health and to the medical profession. The social democrats were dominant in the political landscape in the postwar years, and an approach to drugs as public goods, rather than ordinary commercial entities, increasingly formed a nexus with core values of social democracy. The tight delimitation of the market—excluding products even with proven safety and efficacy—was possible precisely because drugs were not first and foremost regarded as commodities, but had been established as integral to health policy, and later to universal health care. While pointing out these resonances with social democracy, it is also important not to reduce the need clause to its context: the other Nordic countries had similar political situations without similar legislation, and the need clause remained in place under several conservative governments. Minimizing the influence of purely commercial interests in health care was explicitly part of social democratic programs, but the broader notion of treating drugs *not* as ordinary commodities enjoyed wider support.

This approach to drugs also influenced the way the problem of “irrational” use of pharmaceuticals should be handled. In the American context, securing an unbiased knowledge base through the controlled clinical trial was promoted as the solution, whereas in Scandinavia, the emphasis was on alternative ways of promoting health without medicines and prudent use of drugs already on the market. The basis of the public health policy in this period was the expansive definition of health adopted by the World Health Organization (WHO) in 1948, with the same Karl Evang as one of its main architects: “a state of complete physical, mental and social well‐being and not merely the absence of disease or infirmity.”^46^ This holistic definition of health helped the promotion of a drug regime that held that health could be attained by other ways than by consuming medicines, and even be hampered by consuming too much medicine.

The need clause fit nicely with the stated aims of the drug policy at the time, namely, a reliable and cheap provision of drugs for rational use. In Evang's view, the aim of drug control was to “secure the Norwegian population access to *all* valuable and justified drugs. At the same time it must make sure that the number of registered drugs is kept at the lowest possible level that is in accordance with the main goal.”^47^ After his retirement, Evang described the need clause as the single most important part of the Norwegian system of drug regulation—precisely because it allowed the registration authority to pursue such a mission.^48^ ^(p146)^

A low number of drugs on the market presented several advantages. It promoted rational prescribing, since it was easier for physicians to stay up to date with current medical information. It also had very real practical benefits; the physicians could know the drugs they were prescribing better. The aim was to allow the complete drug reference manual list to “fit into the pocket of the white coat.”^49^ ^(p46)^ Multiple names and brands for identical drugs, along with a high number of dosage forms and combinations, would cause unnecessary confusion and risk for doctors, pharmacists, and patients. Maintaining a low number of drugs on the market would further lead to reduced costs related to distribution, storage, and waste when pharmacies would not be obliged to keep long lists of drugs in stock. By allowing a certain number of synonym drugs or brands (the term used at the time was *parallel* drugs), one could also avoid monopolies and obtain competitive pricing. Normally a drug that was cheaper than registered alternatives, but otherwise identical, would be considered as needed. Evang described the aim of drug regulation as ensuring availability of all proven, medically justified drugs, and at the same time keeping the societal costs and the total number as low as possible. The notion of need is clearly one that can be interpreted in different ways, and importantly, it remained unspecified in regulations and official documents throughout the period covered in this article. This meant that the precise number of synonym drugs allowed would be a pragmatic decision that could consider different concerns, such as the importance and level of use of the drug in question or reliable supplies and national self‐sufficiency.

Officials explaining the practice have given different numbers of synonym drugs generally thought to meet the “need,” ranging from two to seven at different times, as well as different therapeutic classes. In 1953 the pharmaceutical control agency stated in a memo the general rule as two to three brands, occasionally up to 11.^50^ Evang, in a textbook on public health, states three to four as the norm, while a later account of the practice for an international audience says five to seven.^20^, ^48^ It was also underlined that this was to be considered merely a rule of thumb.

The need clause clearly enabled the Norwegian health authorities to maintain the number of registered drugs significantly lower than elsewhere.^7^, ^9^, ^51^, ^52^ Specific numbers are not always easily comparable, but Norway and the other Nordic countries clearly had smaller selections of marketed drugs. For instance, a European survey in 1986‐1987 states around 2,000 brands were on the Norwegian market, whereas Germany had 11,000; generally speaking, Western European countries excluding the Nordic region were found to have two to nine times as many chemical entities.^14^ “Lack of need” could typically be the reason given for more than half of rejected applications in Norway in a given year.^11^

## Controversies Over Drug Regulation in a Social Democracy

The years from 1950 to 1970 have been called “social democracy's happy moment” in Scandinavia.^53^ The ideal in health policy was that free health care of excellent quality, including access to the best available treatment, should be provided for all in publicly owned institutions. Shielding or exempting drugs from commercial forces or the logic of the marketplace fits neatly within this frame. The need clause is a clear expression of such a reasoning, but at the time it was far less visible or contested than other aspects of drug policy and its explicitly anticommercial tone. The pharmaceutical industry was initially less concerned with the need clause than it was with regulations favoring local production or compounding in pharmacies. The system introduced in 1928 did not regulate “drugs” in general but “specialties” specifically—in other words, drugs sold in premade packaging and dosage form rather than prepared in the pharmacy. A separate law, exempt from registration and approval procedures, governed this latter form, which was not limited to older drugs, but often was based on ingredients and substances delivered from the pharmaceutical industry, prepared in final form and dosage locally. Thus two parallel sets of regulations applied, and the pharmacy association had been the main driver behind the new regulations, securing important privileges for their own production, such as the normally exclusive right to produce any drug or preparation included in common formularies or pharmacopeia.^19^, ^31^, ^54^

The drug regulation did not remain uncontested, however. First of all, there were repeated protests from the pharmaceutical industry. The Norwegian pharmaceutical industry formed an association (No‐Fa‐Ki) toward the end of World War II, arguing strongly against the government regulations favoring what they described as an outdated relic of the past. In 1946, a long complaint to the Ministry of Health and Social Affairs argued that the legislation was outdated and prevented a necessary development, innovation, and rationalization of drug production. Industrial production was more efficient, would lower the cost of drugs, and would stimulate the growth of a national drug industry, it was argued.^55^

This was certainly a cheerful prospect for the government in a time of scarcity, but there was considerable resistance to such a position from the authorities in the Health Directorate, dominated by doctors and pharmacists. One argument against the “rationalization” through increased industrial production was that this would be a waste of the high level of pharmaceutical competence existing in pharmacies throughout the country, demoting them to dispensers of drugs in ready‐to‐use packaging.^56^ In terms of rationalization, there were more important steps to be made, namely, the continued weeding out of “unnecessary, outdated, ineffective drugs, and limitation of the number of parallel drugs. In this area large sums could be saved, and through this rationalization drug therapy would become more secure and effective.”^57^

The image of the “flood of drugs” continued to be widely used to describe the situation in the postwar era—whether the flood consisted of arcana, foreign specialties, or unnecessary combination drugs or dosage forms, all highly advertised. The pharmacists were regarded by the authorities as a barrier against this threat, by virtue of their professional knowledge and integrity. Evang emphasized the value of what he called the “ethical” attitude developed in the medical and pharmacist professions, in direct opposition to the role of economic concerns.^58^ In contrast to this, the pharmaceutical industry was almost by default under suspicion because of the commercial nature of its activities. This commercial side of the pharmacies, however, was seen as contained within an established system tightly regulating their trade. This system incorporated an understanding of their social responsibility and role in providing health services, such as the obligation to provide all drugs, including the ones that were not profitable. In contrast to the United States, where the influence of the pharmaceutical industry grew steadily in health administration in the postwar years,^59^ in Norway pharmacists had prominent positions in the health administration dealing with drug policy; the industry did not.

The outcome of the industry's complaints has been characterized as a corporatist agreement in which the pharmacies and the domestic industry tentatively distributed their roles within the general national framework of a secure domestic supply and provision of drugs.^31^ Pharmacies and domestic industry had a common interest in reaching an understanding with the authorities: they both feared socialization of their sectors. Socialization was seriously debated at the time, and a law passed by parliament in 1953 established a state monopoly for the wholesale of drugs, the Norwegian Medicinal Depot. Surplus from the monopoly was from the very beginning (in the statutes) determined to be used for social purposes, and the company decided to spend the surplus on knowledge of drug risk, industry‐independent drug information to physicians and patients, as well as means of control policy. In Sweden, pharmacies were nationalized in 1970, while in Norway this was a recurring question until the late 1970s.^31^ State ownership of key drug institutions was, in contrast to the need clause, highly controversial, and it dominated public debate on drug policy for decades.

The pharmaceutical industry had its objections to specific regulations. Its representatives complained and appealed repeatedly to the Specialties Board about rejected applications, but they had very limited grounds to contest the decisions on a legal terrain. The domestic industry was small and far less powerful and influential than has been the case elsewhere, and international companies worked through their representatives in a very small market. Nevertheless, attempts to influence decisions certainly took place, leading, for example, to a decision in 1965 to rule out “personal meetings” between industry and board members or consultants; questions or information had to be submitted in written form.^60^ The nature and extent of contact and affiliation with industry in a very small country was a recurring question for the control agency and its consultants.^61^

Although there was certainly friction between industry and regulatory authority, there was also pragmatic adjustments and seldom direct contestation of the need for a strictly regulated pharmaceutical market. In 1947, while protesting certain measures, the industry association made sure to underline that it found it “evident that the state must secure far‐reaching control with drug production,” that the industry was “naturally fully aware that this is not a common commodity.”^55^ In contrast to many other countries where the introduction of far less comprehensive drug regulation was highly controversial, the basic features of this already established system of drug registration appears to have enjoyed wide support. Even conservative politicians emphasized that there was no disagreement as to the “need for a very strict societal, medical control with the production and distribution of drugs of any kind.”^62^ ^(p892)^ Whereas arguments of professional autonomy hindered procedures for registering new drugs with a central authority in other countries such as Germany, the powerful Norwegian medical profession did not object to government interference in the drugs available for prescription. For instance, when the need clause was initially proposed to be removed in a drug law revision in 1963‐1964, the Norwegian Medical Association did not support this. Medical professionals enjoyed a high level of influence and trust within the health administration. Potential controversies were also evaded by providing a possibility for physicians to obtain unregistered drugs for particular patients through a simple application procedure, an option that was widely used.^63^ This made it easier for the profession to accommodate the strict regulations, and it probably helps explain why we find only occasional protests from physicians in the archives. A 1974 survey indicated that only a small fraction of doctors wanted less involvement by the authorities.^21^ The arguments of “clinical freedom” or professional autonomy were not nearly as important as they have been on the European continent or in the US debates.^43^, ^64^

The 1964 revision updated the regulations in many respects, but it maintained the need clause despite it having been proposed for removal as an outdated form of regulation “introducing purely discretionary judgments on a mercantile level.”^65^ ^(p26)^ Conservative politicians noted disagreement but did not bother taking out formal dissent; instead, they focused on the continued privileges accorded to pharmacies over industry and seized the opportunity to raise yet another debate over the state monopoly on the wholesale of drugs.^66^

## A Flexible Notion of Need: The Case of Cephalosporins in the 1970s

In the context of antibiotic resistance, the need clause provided straightforward grounds for excluding from the market many drugs that were considered to promote resistance. Even when safety and efficacy had been proven, they could be refused with the argument that there was no need for them that could not be met with the proper use of existing drugs. And in the following decades, the threat of antibiotic resistance was frequently brought up in order to argue for a refusal of a new drug. In the 1960s the number of available antibiotics was growing, as was awareness of the problem of microbial resistance development.^67^ A new generation of young doctors was employed as expert consultants on antibiotics by the control agency. They were clearly concerned about the development of antibiotic resistance, and they held strong views on what was to be considered appropriate use.^49^ They formed part of a group we could call the Norwegian version of the “therapeutic reformers.” From positions in the drug agency, the drug wholesale monopoly (with correct drug use part of its mission), the new department of pharmacotherapy at the University of Oslo (funded by surplus from the wholesale drug monopoly), or as infectious disease specialists and microbiologists in hospitals, they could push a similar, restrictive agenda in several arenas: in teaching, in drug selection for hospital formularies, or in the production of industry‐independent drug information circulated to doctors. The latter included a column on recommended therapy in every issue of the journal of the Norwegian Medical Association—and later, a drug manual to serve as an alternative to the ones provided by the industry association. Although the Health Directorate expressed a high level of trust in the medical profession, it simultaneously supported various efforts aimed at countering the influence of industry representatives and advertising on prescription.

In the United States there was public debate following Senator Gaylord Nelson's hearings on the pharmaceutical industry from 1967 to 1976, highlighting the misuse of antibiotics in general and chloramphenicol and fixed‐dose combinations in particular. According to Scott Podolsky, in spite of the fact that the FDA did succeed in removing combination antibiotics from the market, efforts to obtain changes toward rational use of antibiotics “reveal the presence of institutional hesitation and the limits of contemporary aspirations.”^40^ The success of removing these drugs was also followed by their replacement with newer and more expensive drugs, such as cephalosporins and broad‐spectrum penicillins used in a similar manner—an “abject defeat,” according to a disappointed therapeutic reformer in the late 1970s.^68^ In contrast to this experience, proponents for “rational” use of antibiotics in Norway did not have to deal with existing widespread use of combination drugs, and furthermore succeeded in establishing a very strict line on the new antibiotics. From the late 1960s, registration policies were clearly tightened. Limits to prescription (only for use in hospitals, prescribed by specialists, etc.) were attached to the licenses in the sense that restrictive measures at prescription level as well as price would be negotiated at the time of registration. The need clause is a key element in this story, as the notion of need was flexible enough to accommodate much more specific concerns and regulatory agendas than simply limiting the number of synonymous drugs.

The case of cephalosporins provides a good example. Cephalosporins are a group of beta‐lactam antibiotics that inhibit the synthesis of the bacterial cell wall. Manufactured in the United States starting in 1965, there was a subsequent explosion of subclasses throughout the 1970s, developed partly as a response to concerns and perceived markets regarding resistance patterns. The large number of different substances had relatively minor differences in properties.^15^ Hence, the question for the Norwegian registration authority was not one of “parallel” or identical drugs—the typical case that would activate the need clause. Between 1965 and 1971, three different cephalosporins of the “first generation” (cephalotin, cephaloridine, cephalexin) were registered and marketed in five different brand names. In 1979, these were still the only cephalosporins listed in the industry drug manual, and in the meantime, at least 10 different new cephalosporins had been rejected on the basis of a lack of need.^69^

After 1980 a few new cephalosporins were slowly registered (cefuroxime, cefoxitin, cefotaxime), but cephalosporin use remained marginal in Norway.^70^, ^71^ Elsewhere they gained much more widespread use, despite high costs compared to existing alternatives as well as concerns of resistance development.^72^ The need clause was used actively to reject new cephalosporins, and a distinct interpretation of need was established: to be needed, a new drug had to prove *significant advantages* over existing drugs.^69^ This meant a demonstration that the new drug clearly would be the drug of choice in a given situation compared to *any other antibiotic* already on the market, not only compared to near‐equivalent drugs. In the context of a growing awareness of the societal problem of antibiotic resistance, the drug of choice would usually be the (existing) more narrow‐spectrum drugs: good old penicillin was repeatedly referred to as the better choice.^73^ This was the primary reasoning behind skepticism toward registering new and costly cephalosporins, not concerns for a cephalosporin‐specific mechanism of resistance development. “Need” in these assessments would be interpreted explicitly as the ability of the new drug to fill a slot in the therapeutic regime advocated.

This very strict line was not a general approach across all classes of drugs. Rather, “need” could be interpreted this way because the new cephalosporins (along with other new antibiotics) were regarded as problematic—as new, convenient, and broader spectrum, their availability and advertising could lead to prescription where their use was not warranted. They were thus deemed undesirable and superfluous by the expert consultants of the agency. The therapeutic regime they advocated had resistance development as an integral, key concern, and the new cephalosporins could not defend their place in it. According to the agency, the clinical needs were already met, with better‐known and often cheaper and more narrow‐spectrum drugs.

Measures were also taken against cephalosporins and other antibiotics already registered. Since market authorization was tied to approved indications and description in promotional material and drug reference manuals, these could be narrowed down and specified. Registration could also be linked to restriction on prescription to hospitals or specialists. Some of these measures seemed designed to marginalize use of specific drugs—and were made possible by a discretionary assessment of need that remained completely opaque to the pharmaceutical companies and the public alike. It provided a considerable maneuvering space to pursue specific agendas when it came to the preferred therapeutic regime. Decisions could not be appealed on substantial grounds, and when this arrangement had been contested in the revision of the drug law in 1964, Evang had bluntly replied that appeals made little sense: the Specialties Board had already assembled the nation's highest level of medical knowledge, and it would often be impossible to find “over‐expertise” to overrule them.^74^ Rather than appealed decisions in legal terms, complaints and renewed discussions in several rounds in the Specialties Board were common—and here, the flexibility of the need clause provided room for pragmatic adjustments, where several concerns could be weighed against each other. For instance, although the broad‐spectrum penicillins were often treated with similar skepticism as the cephalosporins, one decision concluded with a “no need” rejection, but then explicitly stated it would reconsider if the company lowered the price.^75^ Other examples include complaints and negotiations over approved indications, prescriptions restricted to hospitals, or content of advertising. Initial decisions to restrict prescriptions of antibiotics, such as certain aminoglycosides, to hospitals or to very particular, narrow indications were on several occasions reversed after hearing complaints that this unfairly affected only certain drugs; other times, decisions were upheld.^76^ Although this “maneuvering space” gave experts formidable power over decisions, the flexibility and pragmatic considerations also provided opportunities for pharmaceutical companies to adjust and exert influence over an opaque process. The case of antibiotics shows a strict practice, but the picture could look very different in other classes of drugs, where expert consultants held different opinions or were not in agreement with the board's line, or when other sets of concerns would have to be taken into account.

The Directorate of Health has been described as a stronghold of “expertocracy” in Norwegian postwar history,^77^, ^78^ and in the case of antibiotics, “therapeutic reformers” acting as anonymous consultants certainly had a strong influence on drug policy. This process was shielded by the flexibility of the need clause—and the health director ensured that “need” was not specified or elaborated in guidelines for the registration process, precisely to protect this maneuvering space.^79^ Far from all of the radical measures recommended by the expert consultants were followed, but their arguments were heard and clearly resulted in a very strict practice, aiming to use the registration process as a tool to enforce a usage closer to the therapeutic regime they advocated.

Their evaluations of the new cephalosporins were not necessarily in themselves remarkably strict. Compared to reviews of these drugs published at the same time in reform‐minded journals like *Medical Letter on Drugs and Therapeutics*, or *Drug and Therapeutics Bulletin* (which even recommended amending the British Medicines Act to include something like the need clause^80^), they very often reached the same conclusions and recommendations. These journals often bluntly characterized the drugs as superfluous and said that older alternatives remained preferable. The crucial difference, of course, was that these publications were directed at practicing doctors, hoping to influence their prescribing when the drug was already on the market. The expert assessments discussed here, on the other hand, in reaching similar conclusions, provided the grounds for the Specialties Board to deny the drugs access to the market in the first place. This is probably also why the Norwegian situation was invoked as “the clinical pharmacologist's dream” in a *British Medical Journal* editorial in 1984.^81^ At that time, it would still take four years until a specialty in clinical pharmacology was established in Norway. The Norwegian situation was thus not a product of clinical pharmacologists but of an interdisciplinary group of people consisting of pharmacists and physicians of different specialties. They shared an interest in rational drug use and became highly influential because their views resonated with the general ambitions of the health and drug administrations.

## Epilogue: Demise of the Need Clause and Lessons for Current Debates

The need clause is no longer part of Norwegian drug regulations. It was abandoned in the process of harmonization with European pharmaceutical markets in the early 1990s. The needs approach can still to some extent be said to live on through established patterns of prescription and use, even though the regulatory system is now in line with Europe in general. Is the need clause then little more than a national peculiarity—simply a footnote in the history of drug regulation? Our argument is that it merits renewed attention: along with the wider system it was part of, it is relevant to important current debates in the global politics of pharmaceuticals. It has recently again been brought up as a point of reference and contrast—in terms of controlling overall cost, steering pharmaceutical research toward genuine therapeutic advances, or better meeting global health needs.^4^, ^5^, ^8^

In fact, the terms of this discussion clearly resonate with controversies surrounding the need clause in the years before it was taken out of the regulatory system. First, the need clause, along with the overall “Nordic” regulatory approach it was part of, came to play an important role in international controversies over pharmaceutical policy from the late 1970s. This was particularly so with the World Health Organization's Essential Drugs program, which aimed to define a core selection of indispensable drugs that should be universally available.^82^, ^83^, ^84^ In many developing countries in particular, with limited capacities for controlling the pharmaceutical market, staggering numbers of products were marketed.^85^ They were often outdated, of inferior quality, and heavily advertised, and drugs accounted for major parts of health expenditure. Norway was a frequently cited example of a country where only a limited number of drugs were available, without detrimental effects. Although there were marked differences between the Nordic countries in terms of their specific drug regulations, they also cooperated closely.^21^ They formed a “Nordic bloc” in alliance with developing countries and sometimes consumer activists in the often highly controversial struggles over the Essential Drugs program, which became an arena for wider conflicts surrounding the pharmaceutical industry. As one sought to make the principles of the Essential Drugs approach valid also for industrialized countries, the need clause was prominently placed within more general discussions on measures to ensure a more rational use of drugs—with the clear implication that this would mean much stronger regulation of the pharmaceutical industry globally. This controversy culminated in the 1985 WHO conference in Nairobi, titled “The Rational Use of Drugs,” where the Norwegian example was repeatedly debated and highly contested.^86^ More generally, the Nordic countries cooperated and met regularly to coordinate positions in the WHO, and they advocated drug reforms along the lines of their own systems.^87^ They confidently argued that the world could learn from their “social” and “rational” approach, and Norwegian delegates explicitly linked the need clause with the Essential Drugs program and a rational drug policy.^88^

The wider agenda for rational drug policies within the framework of the WHO included other closely related initiatives in which Nordic participants and the Nordic example continued to play a prominent role, such as the drug utilization studies, explicitly aimed at rationalizing drug use and informing drug selection and partly originating from the unique availability of statistical material from the Norwegian wholesale monopoly.^85^, ^89^, ^90^, ^91^, ^92^ This is an interesting story that far exceeds the scope of this paper, but it is safe to say that by the second half of the 1980s, the Nordic “rational,” health‐centric approach was losing ground both internationally and at home. The need clause had depended simultaneously on a strong position and autonomy of medical expertise within national health authorities, and on the subordination of principles of market regulations to more general public health concerns. Both of these factors increasingly came under pressure beginning in the late 1980s, which brought harmonization with the European pharmaceutical market and the gradual abandonment of many of the regulations peculiar to the Nordic countries.

Although Norway did not join the European Commission after a referendum in 1994, it did join the internal market by signing the European Economic Area (EEA) agreement in 1992. The process involved wide‐ranging harmonization of national regulations, and pharmaceuticals was a particularly complex and difficult field.^93^, ^94^, ^95^ The wholesale monopoly and the need clause were both deemed incompatible with entry into the common market and were removed from the national drug policy after negotiations.^18^, ^96^, ^97^, ^98^ The need clause had also been a contentious point in earlier efforts for closer Nordic integration of drug registration procedures in the 1960s and 1970s and again as a problematic “trade barrier” within the Nordic countries in the late 1980s.

In the former case, the Norwegian health authorities had actually signaled a willingness to give up the need clause in order to achieve a common Nordic registration process.^21^ In the latter, however, the need clause was defended as a crucial element in the national policy.^99^ In the meantime, the Norwegian drug regulatory authorities had grown used to defending their own system as the best or strictest in the world. But while maintaining a confident and assertive position abroad, the “medicracy”—or the strong position of doctors and medical knowledge in the health bureaucracy—was losing ground at home. Controversial restructuring processes in 1983 and 1992 demonstrated a loss of the Directorate of Health's autonomous position and grip on health policy,^100^ and with it, perhaps, its ability to protect “national peculiarities” under its authority, like drug policies.

Health director Torbjørn Mork vigorously—and increasingly in public—tried to defend the established policies against his superiors in political leadership as well as against other governmental branches, such as pricing authorities. The conflict over drugs as a question of either public health or commercial policy clearly echoes Evang's earlier, successful initiative to secure control over drug policy against the ministries of industry and commerce. The need clause, Mork argued in a letter to the minister of social affairs, was a “foundational element” in the national drug policy, regarded by doctors as an important means of maintaining high scientific standards and rational use.^101^ An appended background note further laid out the virtues of the need clause: it was in large part responsible for the small number of marketed drugs—one‐tenth of some other European countries—it was completely in line with the WHO's guidelines for rational drug policies, and it had been a role model internationally.

The minister initially supported the position completely, but already the following year, when the government was preparing for negotiations for entry into the European common market, signals were clear. The need clause and wholesale monopoly were regarded as incompatible with European harmonization, and after decisions in the cabinet, the directorate was instructed to develop transition plans.^102^ The directorate reluctantly complied but kept arguing strongly against the abandonment of the need clause.

Mork later went on to publicly accuse his own political superiors of using the Europeanization process as an excuse to go further than what was actually needed to “harmonize” drug regulation, and in the EEA negotiation process, documentation of these points remained hidden from public view; even opposition politicians could not obtain access after direct requests in parliament.^103^ The removal of the need clause increased the number of drugs available but never led to a “flood of drugs,” as conditions for approval similar to the other Nordic countries remained in place.^96^ In fact, the guidelines developed through the Nordic Council on Medicines collaboration from 1975 onward were crucial in the formulation of the harmonized European registration procedures, where whole segments were taken from the Nordic guidelines.^18^ This process, as well as the formation of the European Medicines Agency and regulatory network of national authorities in the 1990s, has been important in standardizing evaluation of medicines,^104^ but the influence from the Nordic countries did not include wider societal concerns as part of registration criteria. In sum, the Norwegian drug regulation of the 1990s saw a transition to the market‐centric system characteristic of Europe.^98^ This can clearly be seen in light of more general movements in the 1980s and 1990s toward the dismantling of state regulation in favor of market‐based principles of governance and creation of more unified markets. However, the need clause was not simply a reflection of these processes or something that “inevitably” belonged to a past regulatory regime. Rather, its removal formed part of wider negotiations and bargaining over trade agreements—effectuated by a labor government—at a time when it still served as a model for other countries’ regulatory authorities.

When discussing the need clause, it is important to underline that considerations of need are not as unique as it may seem. Similar considerations have been and still are at work elsewhere: in hospital formularies, in various reimbursement schemes, and in the “essential drugs” concept, selections are made among a wider array of drugs, incorporating larger social, economic, and other considerations.^8^, ^91^, ^105^, ^106^ The director of the control agency, Magne Halse, made a similar point in 1980, when he described the criterion of need first and foremost as a *way of thinking*, not exclusive to the Norwegian legislation, and further, that the limitations it entailed were perceived “as part of the work to promote a rational therapeutics.”^107^ ^(p128)^ In other words, rather than simply being a peculiarity of the Norwegian system, the need clause was a clear‐cut example of principles that are also at work elsewhere in the economy of pharmaceuticals. The crucial difference, of course, is that drug selection takes place at the level of market access, and thus directly creates the universe of available drugs, rather than indirectly influencing their use. But more general effects are also important to take into account. As Daniel Carpenter noted in his analysis of the FDA, a “conceptual” facet of regulatory power denotes how expectations or requirements can shape “patterns of thought” far beyond formal decisions within the institution.^25^ ^(p64)^ The need clause functioned in a similar manner. It found resonance with and provided reinforcement and authority to a “way of thinking” about drugs—in formularies or prescribing patterns—far beyond the specific area of market authorization.

This way of thinking—which tended to describe itself as almost inseparably “social” and “rational”—in fact addressed all three parts of the commonly identified challenges involved in curbing global antibiotic resistance: providing universal access, limiting excess use, and incentivizing the development of new antibiotics.^108^ Access to all valuable and medically justifiable drugs for the entire population was, as quoted above, Evang's way of describing the core aim of drug regulation. This fundamental orientation toward universal health needs further explains why Evang deemed the need clause to be a crucial regulatory tool, and why the Essential Drugs program's aims initially had a strong affinity with such a model.

On the side of appropriate use: As we have seen, the Norwegian system was always addressed toward appropriate or “rational” use, not primarily toward safety. This way of thinking of the regulatory system as a way to promote appropriate use through forming social norms is an aspect we notice is often absent from the current debates on drug regulation. As we have seen, the need approach of the Norwegian drug regulatory system did not only aim to reduce the total number of available drugs; it also wanted to influence prescription in the direction of appropriate use. For antibiotics, this has clearly affected the patterns of use and habits of prescription favorably—patterns that to a large extent remain in place to this day.

A need approach also indirectly addresses the current strong disincentives for profit‐driven companies to invest in research for new antibiotics, for which use must be minimized. The need clause has recently been discussed in terms of the costs of pharmaceuticals, and more specifically as a solution to the structural problems of pharmaceutical innovation to a large extent being directed toward classes of drugs that are highly profitable but where very little therapeutic advance is achieved.^4^, ^5^, ^6^ A need approach offers a radically different set of incentives for research, by only allowing market access to new drugs that offer genuine therapeutic advance or added therapeutic value in today's terms. As Graham Dukes has commented on the need clause, “had it been employed in more countries this approach could well have promoted a shift from ‘me‐too’ investment to true innovation.”^109^ ^(p258)^ By incorporating wider concerns at the initial point of market access, a need clause could be a powerful measure—among several recently suggested^110^, ^111^—to steer pharmaceutical innovation in the direction of genuine, societal health needs. Far from being simply a marginal concern, the need clause is rather an example that goes to the heart of long‐standing debates over drug regulation internationally, the inclusion of relative efficacy requirements, and the added therapeutic value of new drugs.^4^, ^94^, ^112^

Perhaps the most general point to be made here is that the Norwegian system represented a clear‐cut example of a policy that did not regulate drugs primarily as commodities. It subordinated this aspect of the drug to its function from a public health perspective.^98^ As such, it represented a striking alternative in the international controversies over pharmaceutical policies in the 1970s and 1980s, along with the other Nordic countries sharing this basic orientation. Today, when a range of global initiatives are under way looking for new ways to incentivize pharmaceutical companies to invest in antibiotic research, to control overuse, and to secure access for those in need, the abandoned need clause merits a closer look. The phrase “less is more,” often used for Scandinavian design with its focus on sustainable, practical, and affordable products accessible to all users, could also be fitting for a drug policy aiming at sustainability and the public good.

## Archives consulted

Parts of the historical archives of the current Norwegian Medicines Agency (NoMA) are not yet indexed. All of the material cited from here is categorized as “Helsedirektørens sakkyndige råd”/“Spesialitetsnemnda,” containing minutes, documents and appendices from the meetings of the Health Director's Expert Council/the Specialties Board.

Archives of the Norwegian Medicines Agency (NoMA)

Archives of the Norwegian Health Directorate (National Archives of Norway; NAN)

Archives of the Specialties Control (National Archives of Norway; NAN)

Archives of the Norwegian Board of Health Supervision
